# Exercise Reduces H3K9me3 and Regulates Brain Derived Neurotrophic Factor and GABRA2 in an Age Dependent Manner

**DOI:** 10.3389/fnagi.2021.798297

**Published:** 2021-12-14

**Authors:** Andra Ionescu-Tucker, Christopher W. Butler, Nicole C. Berchtold, Dina P. Matheos, Marcelo A. Wood, Carl W. Cotman

**Affiliations:** ^1^Department of Neurobiology and Behavior, University of California, Irvine, Irvine, CA, United States; ^2^Institute for Memory Impairments and Neurological Disorders, University of California, Irvine, Irvine, CA, United States; ^3^Center for the Neurobiology of Learning and Memory, University of California, Irvine, Irvine, CA, United States

**Keywords:** epigenetics, exercise, H3K9me3, ETP69, BDNF, aging, hippocampus

## Abstract

Exercise improves cognition in the aging brain and is a key regulator of neuronal plasticity genes such as BDNF. However, the mechanism by which exercise modifies gene expression continues to be explored. The repressive histone modification H3K9me3 has been shown to impair cognition, reduce synaptic density and decrease BDNF in aged but not young mice. Treatment with ETP69, a selective inhibitor of H3K9me3’s catalyzing enzyme (SUV39H1), restores synapses, BDNF and cognitive performance. GABA receptor expression, which modulates BDNF secretion, is also modulated by exercise and H3K9me3. In this study, we examined if exercise and ETP69 regulated neuronal plasticity genes by reducing H3K9me3 at their promoter regions. We further determined the effect of age on H3K9me3 promoter binding and neuronal plasticity gene expression. Exercise and ETP69 decreased H3K9me3 at BDNF promoter VI in aged mice, corresponding with an increase in BDNF VI expression with ETP69. Exercise increased GABRA2 in aged mice while increasing BDNF 1 in young mice, and both exercise and ETP69 reduced GABRA2 in young mice. Overall, H3K9me3 repression at BDNF and GABA receptor promoters decreased with age. Our findings suggest that exercise and SUV39H1 inhibition differentially modulate BDNF and GABRA2 expression in an age dependent manner.

## Introduction

Exercise has been shown to improve cognition in the aged brain across a range of clinical and animal studies (van Praag et al., 2005; Kirk-Sanchez and McGough, 2014; Snigdha et al., 2014). Underlying these improvements are changes to the expression of genes related to neuronal plasticity. Our lab was the first to show a dose-dependent effect of exercise on hippocampal brain derived neurotrophic factor (BDNF), which is critical for memory, neuron survival and brain plasticity (Neeper et al., 1995; Berchtold et al., 2002; Cotman and Berchtold, 2002). Recent studies suggest that exercise changes gene expression via epigenetic mechanisms, which are also critical contributors to age-related cognitive decline (Lardenoije et al., 2015; Fernandes et al., 2017). A dynamic repressive mark is trimethylated histone 3, lysine 9 (H3K9me3), which is associated with age-related gene repression and regulates genes critical for cognitive function (Sedivy et al., 2008; Villeneuve et al., 2010; Snigdha et al., 2016).

Our lab has focused on elucidating the role of H3K9me3 in cognitive function. We first determined that H3K9me3 is elevated in the hippocampi of aged mice. To investigate if H3K9me3 is involved in cognitive decline, we inhibited the histone methyltransferase (SUV39H1) that catalyzes H3K9 trimethylation. We used a selective SUV39H1 inhibitor called ETP69, a chaetocin A analog developed by Dr. Larry Overman (Baumann et al., 2015). Treatment with ETP69 improved the performance of aged mice in object location memory (OLM) and fear conditioning tasks. ETP69 also increased hippocampal spine density as measured by Golgi staining and flow cytometry. Importantly, ETP69 treatment before or after acquisition of an OLM task increased total BDNF levels and decreased H3K9me3 at the BDNF 1 promoter (Snigdha et al., 2016). These findings demonstrate that H3K9me3 leads to memory decline, reduces synaptic plasticity, and represses BDNF expression in aged mice. While this study examined the relationship between H3K9me3 and memory, the effects of exercise on H3K9me3 gene repression have not been investigated. Further research is needed to determine if exercise-induced increases in neuronal gene expression correlate with a reduction in H3K9me3 at promoter regions.

Studies have begun to examine how exercise affects the regulation of neuronal plasticity genes such as BDNF, which enhances learning and memory (Cotman and Berchtold, 2002; Intlekofer et al., 2013). BDNF is comprised of a variable 5′ (exon I, IIa/IIb/IIc, III, IV, V) end and a common 3′ (exon VI) end (Adlard et al., 2005; Aid et al., 2007). We have shown that the degree of exercise is proportional to hippocampal BDNF expression in young mice and rats (Neeper et al., 1995; Adlard et al., 2004). However, more than 1 week of exercise does not significantly increase BDNF in mature (15 month old) and aged (24 month old) mice, suggesting that BDNF expression is impaired in older mice (Adlard et al., 2005). Reduced GABA signaling may play a role in the age-associated decline of BDNF. GABA B receptors phosphorylate α-CAMKII, involved in BDNF release, and BDNF secretion triggered by GABA B receptors increases levels of GABA A receptors (Kolarow et al., 2007; Fiorentino et al., 2009; Porcher et al., 2018). GABA A receptor activation also increases BDNF secretion via an ERK dependent pathway (Brady et al., 2018). GABA receptors are thus involved in the regulation of BDNF signaling, and GABA signaling is also reduced in the hippocampus with age (Stanley and Shetty, 2004; McQuail et al., 2015). We recently found that exercise correlates with the expression of 8 GABA genes in the aged hippocampus, including GABA B receptor 1 (GABBR1) and the alpha 2 subunit of the GABA A receptor (GABRA2) (Berchtold et al., 2019). H3K9me3 may play a role in the repression of GABA receptors as well as BDNF. A study of postmortem cortical samples showed an increase in H3K9me3 at the promoters of BDNF, GABBR1 and GABRA2 in Alzheimer’s diseased brains (Lee et al., 2020). The parallels between GABA receptor and BDNF expression with age and exercise suggests that GABA signaling may be involved in exercise-induced BDNF regulation.

In this study, we examined how exercise, SUV39H1 inhibition and age affect H3K9me3 promoter repression and expression of BDNF and GABA receptors. We first separated aged and young mice into sedentary, exercised and ETP69 treatment groups. We then measured levels of H3K9me3 at the promoters of BDNF transcripts I, IV, and VI, and at the promoters of GABBR1 and GABRA2. Lastly, we quantified transcript mRNA to determine the correlation between promoter H3K9me3 levels and total gene expression. Exercise and ETP69 reduced H3K9me3 at BDNF promoter VI in aged mice, corresponding with an increase in BDNF VI expression with ETP69. In young mice, exercise increased BDNF I expression, suggesting an age-dependent change in BDNF regulation. Exercise increased GABRA2 expression in aged mice while both exercise and ETP69 reduced GABRA2 expression in young mice. Overall, H3K9me3 repression at BDNF and GABA receptor promoters decreased with age. Our findings suggest that exercise and SUV39H1 inhibition differentially modulate BDNF and GABRA2 expression in an age dependent manner.

## Materials and Methods

### Animals

All experiments were conducted in accordance with the National Institutes of Health guidelines for animal care and use, and were approved by the Institutional Animal Care and Use Committee of the University of California, Irvine. Male 18 month old and 3 month old C57BL/6J mice (*n* = 21 for each age; Jackson Laboratory) were individually housed with food and water *ad libitum*, and allowed 1 week acclimation to the vivarium prior to experiments. Lights were maintained on a 12 h light–dark cycle. Mice were sacrificed by carbon dioxide gas and cervical dislocation, and brains were rapidly frozen on isopentane cooled in liquid nitrogen. 1 mm punches of the dorsal hippocampus were collected from 500 μm slices.

### Exercise and ETP69 Treatment

All mice were single housed in either standard cages (sedentary and ETP69 injected mice) or cages with a running wheel (exercised mice). Mice were single housed due to the design of the running wheel apparatus, which allows for only a single animal per cage. Single housing also gave each animal free and equal access to a running wheel (Adlard et al., 2011). Exercise cages consisted of 24 cm × 35 cm × 20 cm clear plastic, containing a running wheel 40 cm in circumference, 12.7 cm diameter (Lafayette). Aged exercised mice had access to a running wheel for 6 weeks and young exercised mice had access to a running wheel for 4 weeks. 3 weeks was previously shown to improve learning and memory in young mice, and aged mice were exercised for longer to compensate for a reduction in running intensity with age (Adlard et al., 2005; Intlekofer et al., 2013). Mice were i.p. injected with 10 mg/kg ETP69 dissolved in 50% weight/volume β cyclodextrin 24 h prior to sacrifice.

### Chromatin Immunoprecipitation

ChIP was performed as described previously based on the protocol from the Millipore Magna ChIP kit (Malvaez et al., 2011; Rogge et al., 2013). Tissue was cross-linked with 1% formaldehyde (Sigma), lysed and sonicated, and chromatin was immunoprecipitated overnight with 2 μL of H3K9me3 (ab8898) or 5 μL of anti-mouse IgG (negative control, Millipore). The immunoprecipitate was collected using magnetic protein A beads (Millipore). After washing, chromatin was eluted from the beads and reverse cross-linked in the presence of proteinase K before column purification of DNA. BDNF, GABBR1, and GABRA2 promoter enrichment in ChIP samples was measured by quantitative real-time PCR using the Roche 480 LightCycler and SYBR green. Primer sequences for BDNF promoters were taken from Intlekofer et al. (2013) and GABA receptor primers were designed by the Primer-BLAST program (Supplementary Table 1). Five microliters of input, anti-H3K9me3, or anti-mouse IgG immunoprecipitate from mice in each condition were examined in duplicate. To normalize ChIP-qPCR data, we used the percent input method. The input sample was adjusted to 100% and both the IP and IgG samples were calculated as a percent of this input using the formula: 100 × AE^[adjusted input –Ct (IP)]. An in-plate standard curve determined amplification efficiency (AE).

### Quantitative qRT-PCR

qRT-PCR was performed as previously described (Rogge et al., 2013). RNA was isolated from punches using an RNA Easy Mini Kit (Qiagen) and cDNA was created using the High-Capacity cDNA Reverse Transcription Kit with RNase Inhibitor (Thermo Fisher Scientific). Primer and probe sequences were designed by Integrated DNA Technologies and are listed in Supplementary Table 2. All probes were conjugated to the dye FAM except for GAPDH, which was conjugated to the dye HEX. Samples were measured in duplicate using the Roche 480 LightCycler and Gene Expression Master Mix (Integrated DNA Technologies). All values were normalized to GAPDH expression levels. Analysis and statistics were performed based on the Pfaffl method (Pfaffl, 2001).

### Statistical Analysis

Results were analyzed using one way ANOVA, Welch’s ANOVA, or two way ANOVA, followed by *post hoc* analysis using Prism software. In all cases, *p* ≤ 0.05 was considered significant. Excel was used for percent input analysis of ChIP data and Pfaffl analysis of qRT-qPCR data.

## Results

### ETP69 and Exercise Reduce H3K9me3 at Brain Derived Neurotrophic Factor Promoter VI in Aged Mice

**FIGURE 1:**
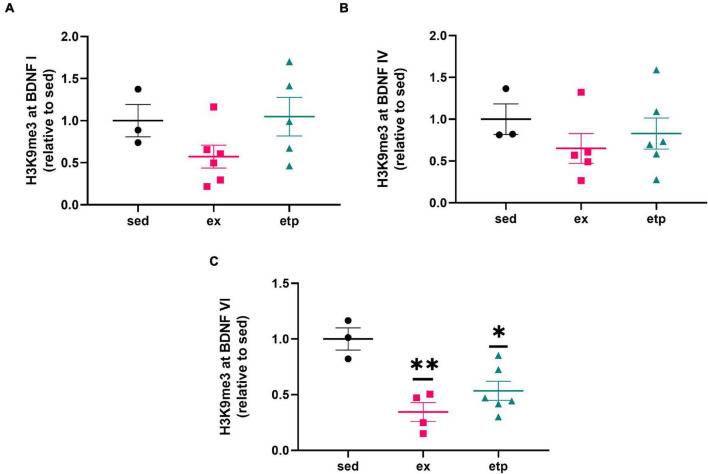
Exercise and ETP69 reduce H3K9me3 at BDNF promoter VI in aged mice. Exercise and ETP69 treatment significantly reduce H3K9me3 expression at BDNF promoter VI **(C)**, but not at promoter I (**A**, One way ANOVA, *p* = 0.16) or IV (**B**, One way ANOVA, *p* = 0.17). **(C)** One way ANOVA **p = 0.0036; Dunnett’s *post hoc* test (**p = 0.0021 exercise vs. sedentary; *p = 0.011 ETP69 vs. sedentary).

We first investigated if ETP69 and exercise affected H3K9 trimethylation levels at BDNF promoter regions. We examined BDNF I, IV, and VI as these regulatory exons are highly expressed in the hippocampus (Aid et al., 2007). Our lab previously found that ETP69 treatment before or after the acquisition phase of an OLM task improved performance in aged mice and reduced H3K9me3 at the BDNF I promoter (Snigdha et al., 2016). Building on this study, we investigated if ETP69’s effect on BDNF repression was dependent on neuronal stimulation or a behavioral task. Treating sedentary, non-behaving aged mice with ETP69 reduced H3K9me3 at BDNF promoter VI instead of promoter I (Figure 1, One way ANOVA **p = 0.0036; Dunnett’s *post hoc* test, *p = 0.011 ETP69 vs. sedentary). This suggests that the neuronal activity caused by an OLM task changes the regulation of BDNF promoter regions.

**FIGURE 2:**
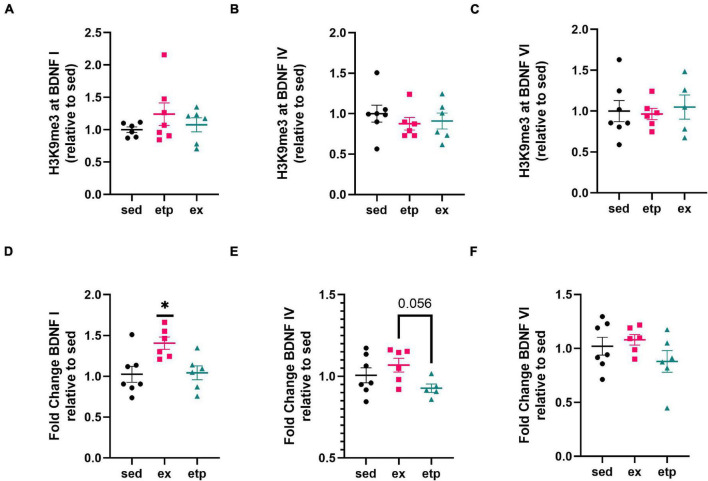
Exercise increases BDNF I expression in young mice. **(A)** H3K9me3 at BDNF I promoter, one way ANOVA, *p* = 0.41. **(B)** H3K9me3 at BDNF IV promoter, one way ANOVA, *p* = 0.6. **(C)** H3K9me3 at BDNF VI promoter, one way ANOVA, *p* = 0.89. Exercise increases BDNF I expression **(D)** but not BDNF IV **(E)** or BDNF VI **(F)** expression relative to sedentary conditions. **(D)** One way ANOVA, *p = 0.012; Tukey’s *post hoc* test, *p = 0.018 sed vs. ex, *p = 0.03 ex vs. etp. **(E)** Welch’s ANOVA, *p = 0.049; Dunnett’s *post hoc* test, *p = 0.056 ex vs. etp. **(F)** One way ANOVA, *p* = 0.24.

We also explored the effect of physical activity on H3K9 trimethylation at BDNF promoter regions, as BDNF production is increased in young but not aged mice after long term exercise (Neeper et al., 1995; Adlard et al., 2004, 2005). Our lab previously found that exercise increases H4K8ac at BDNF I and IV promoters in young mice, suggesting that exercise may also influence H3K9me3 levels at these promoters (Intlekofer et al., 2013). Exercise reduced H3K9me3 levels at the BDNF VI transcript in aged mice (Figure 1C; **p = 0.0021 exercise vs. sedentary), displaying a specific and parallel effect of exercise and ETP69 on BDNF promoters. Surprisingly, neither ETP69 nor exercise changed H3K9me3 at BDNF promoter transcripts in young mice (Figures 2A–C), suggesting that the stability of promoter repression decreases with age.

### Exercise and ETP69 Differentially Regulate Brain Derived Neurotrophic Factor Exon Expression in Young and Aged Mice

**FIGURE 3:**
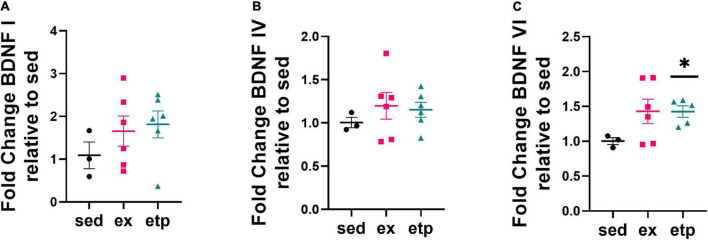
ETP69 increases BDNF VI expression in aged mice. **(C)** Welch’s ANOVA, **p = 0.007; Dunnett’s *post hoc* test, etp vs. sed *p = 0.012. Exercise and ETP69 did not affect levels of BDNF I **(A)** or BDNF IV **(B)** in aged mice. **(A)** One way ANOVA, *p* = 0.43. **(B)** One way ANOVA, *p* = 0.63.

To determine if a reduction in H3K9me3 is associated with increased BDNF expression, we measured BDNF exon mRNA levels in aged and young mice. Reduced H3K9me3 at BDNF promoter VI correlated with an increase in BDNF VI expression in aged mice treated with ETP69, but not aged exercised mice (Figure 3C; Welch’s ANOVA **p = 0.007; Dunnett’s *post hoc* test, etp vs. sed *p = 0.012). There was no change in BDNF I or IV expression with ETP69 or exercise in aged mice (Figures 3A,B). This finding is supported by previous work from our lab showing that exercise did not increase expression of BDNF exons I, IV, or VI in the aged mouse hippocampus (Adlard et al., 2005). These results suggest that BDNF VI levels are regulated by a direct reduction in H3K9me3 repression in aged mice. In contrast, exercise activates multiple signaling pathways that modulate BDNF expression, and its reduction of H3K9me3 does not drive an increase in exon VI expression.

Neither exercise nor ETP69 affected H3K9me3 binding to BDNF promoters in young mice (Figures 2A–C), yet exercise significantly increased BDNF I expression (Figure 2D; One way ANOVA, *p = 0.012; Tukey’s *post hoc* test, *p = 0.018 sed vs. ex, *p = 0.03 ex vs. etp). Exercise significantly elevated BDNF IV levels in comparison to ETP69 treatment, but failed to reach significance relative to sedentary controls (Figure 2E; Welch’s ANOVA, *p = 0.049; Dunnett’s *post hoc* test, *p = 0.056 ex vs. etp). Exercise and ETP69 did not increase BDNF VI levels in young mice (Figure 2F; One way ANOVA, *p* = 0.24). This is in accordance with previous findings that exercise induces BDNF I and IV expression but not BDNF VI expression in young mice after 3 weeks of exercise (Intlekofer et al., 2013). Exercise has a greater effect on BDNF exon expression in young mice than in aged mice, suggesting that BDNF induction by exercise is less prevalent with age.

### Exercise and ETP69 Modulate GABRA2 Expression in an Age Dependent Manner

**FIGURE 4:**
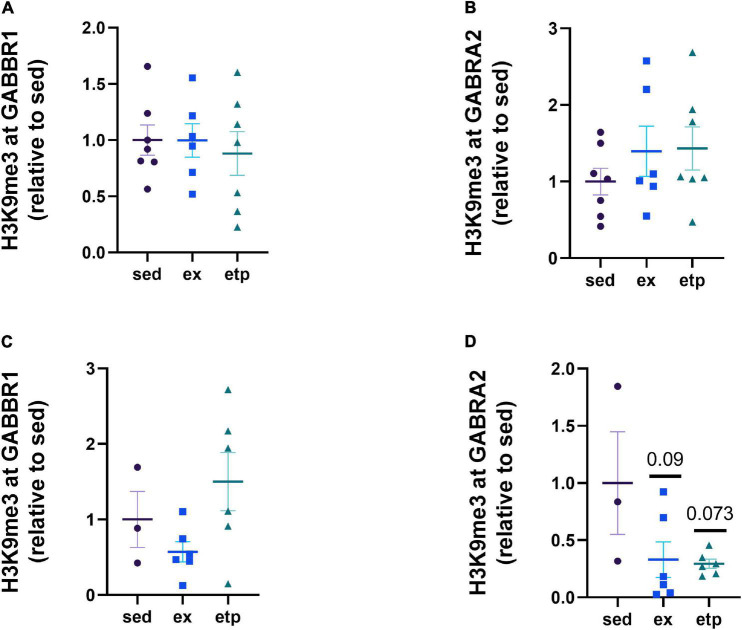
Exercise and ETP69 do not change H3K9me3 at GABA receptor promoters in young and aged mice. **(A)** H3K9me3 at GABBR1 promoter in young mice, one way ANOVA, *p* = 0.89. **(B)** H3K9me3 at GABRA2 promoter in young mice, one way ANOVA, *p* = 0.44. **(C)** H3K9me3 at GABBR1 promoter in aged mice, one way ANOVA, *p* = 0.11. **(D)** H3K9me3 at GABRA2 promoter in aged mice, one way ANOVA, *p* = 0.066; Dunnett’s *post hoc* test, *p* = 0.09 sed vs. ex, *p* = 0.073 sed vs. etp.

We next investigated how the repression and expression of GABA receptors correlate with BDNF, as GABA receptor activation increases BDNF signaling (Kolarow et al., 2007; Fiorentino et al., 2009; Brady et al., 2018; Porcher et al., 2018). We previously found that both BDNF and GABA receptor expression are regulated by exercise, suggesting that GABA receptors may be involved in regulating exercise-induced BDNF expression (Adlard et al., 2004; Berchtold et al., 2019). We first examined if H3K9me3 levels at the GABBR1 and GABRA2 promoters were changed by exercise and ETP69. Promoter repression was not significantly different from sedentary mice in either young or aged animals (Figure 4). However, there was a non-significant reduction in H3K9me3 at GABRA2 in aged animals with exercise and ETP69 (Figure 4D; One way ANOVA, *p* = 0.066; Dunnett’s *post hoc* test, *p* = 0.09 sed vs. ex, *p* = 0.073 sed vs. etp). These results suggest that GABA receptor promoters are resistant to epigenetic changes by exercise and global reductions in H3K9me3.

**FIGURE 5:**
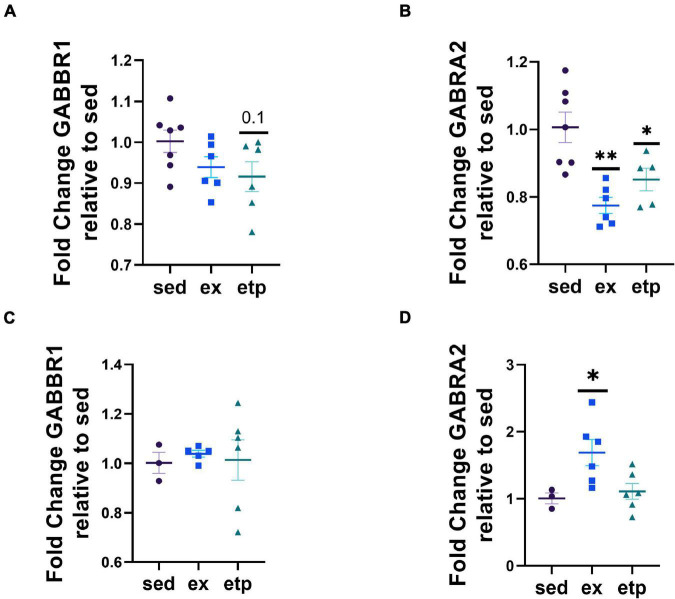
Exercise and ETP69 affect GABRA2 expression in an age-dependent manner. **(A)** One way ANOVA, *p* = 0.13; Tukey’s *post hoc* test, *p* = 0.12 sed vs. etp. Exercise and ETP69 reduced GABRA2 expression in young mice. **(B)** One way ANOVA, **p = 0.0012; Tukey’s *post hoc* test, **p = 0.001 sed vs. ex, *p = 0.28 sed vs. etp. Exercise and ETP69 did not affect levels of GABBR1 in aged mice. **(C)** One way ANOVA, *p* = 0.93. Exercise increased GABRA2 expression in aged mice. **(D)** One way ANOVA, *p = 0.024; Tukey’s *post hoc* test, ex vs. sed *p = 0.052, ex vs. etp *p = 0.044.

We next asked if exercise and ETP69 changed GABA receptor expression levels and found that the direction of change was highly dependent on mouse age. Exercise significantly increased GABRA2 expression in aged mice (Figure 5D; One way ANOVA, *p = 0.024; Tukey’s *post hoc* test, ex vs. sed *p = 0.052, ex vs. etp *p = 0.044). In contrast, exercise and ETP69 reduced GABRA2 expression in young mice (Figure 5B; One way ANOVA, **p = 0.0012; Tukey’s *post hoc* test, **p = 0.001 sed vs. ex, *p = 0.28 sed vs. etp). ETP69’s effect on GABRA2 is surprising considering it does not reduce H3K9me3 at its promoter, suggesting that a global reduction in H3K9me3 activates a pathway that decreases inhibitory signaling. The exercise-induced increase in GABRA2 in aged mice is supported by our research in aged postmortem brains, suggesting that the need for inhibitory signaling increases in the aged brain (Berchtold et al., 2019). There was no treatment effect on GABBR1 expression, although there was a non-significant decrease with ETP69 in young mice (Figure 5A, One way ANOVA, *p* = 0.13; Tukey’s post hoc test, *p* = 0.12 sed vs. etp; Figure 5C, One way ANOVA, *p* = 0.93). Therefore, exercise and ETP69 regulate GABRA2 expression independently of promoter H3K9me3 in an age-dependent manner.

### H3K9me3 Repression of Brain Derived Neurotrophic Factor and GABA Receptors Decreases With Age

**FIGURE 6:**
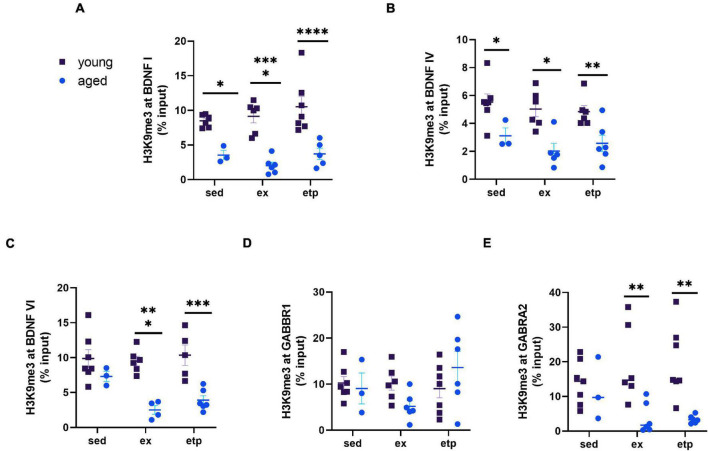
H3K9me3 repression of BDNF and GABA receptors decreases with age. Comparison of ChIP% input data from young and aged mice. **(A)** H3K9me3 levels at the BDNF 1 promoter were significantly greater in young mice for all three conditions (sedentary, exercise, ETP69). Two way ANOVA main effect of age *F*(1,27) = 56.73, ****p < 0.0001. No significant effect of treatment [*F*(2,27) = 1.369, *p* = 0.27] or interaction [*F*(2,27), *p* = 0.58]. Sidak’s multiple comparisons test sed young vs. aged *t*(7) = 3.03, *p = 0.016; ex young vs. aged *t*(10) = 5.33, ****p < 0.0001; etp young vs. aged *t*(10) = 5.041, ****p < 0.0001. **(B)** H3K9me3 levels at the BDNF IV promoter were significantly greater in young mice for all three conditions. Two way ANOVA main effect of age *F*(1,27) = 29.23, ****p < 0.0001. No significant effect of treatment [*F*(2,27) = 0.41, *p* = 0.41] or interaction [*F*(2,27) = 0.24, *p* = 0.79]. Sidak’s multiple comparisons test sed young vs. aged *t*(8) = 2.67, *p = 0.037; ex young vs. aged *t*(9) = 3.78, **p = 0.0024; etp young vs. aged *t*(10) = 2.99, *p = 0.017. **(C)** H3K9me3 levels at the BDNF VI promoter were significantly greater in young exercised and ETP69 treated mice. Two way ANOVA main effect of age *F*(1,25) = 34.9, ****p < 0.0001. No significant effect of treatment [*F*(2,25) = 2.23, *p* = 0.13] or interaction [*F*(2,25) = 2.552, *p* = 0.098]. Sidak’s multiple comparisons test sed young vs. aged *t*(8) = 1.54, *p* = 0.36; ex young vs. aged *t*(8) = 4.49, ****p = 0.0004; etp young vs. aged *t*(9) = 4.41, ****p = 0.0005. **(D)** H3K9me3 levels at the GABBR1 promoter were not significantly different between young and aged mice. Two way ANOVA no significant effect of age [*F*(1,29) = 0.09, *p* = 0.77], treatment [*F*(2,29) = 1.48, *p* = 0.24] or interaction [*F*(2,29) = 2.65, *p* = 0.088]. **(E)** H3K9me3 levels at the GABRA2 promoter were significantly greater in young exercised and ETP69 treated mice. Two way ANOVA main effect of age *F*(1,29) = 17.28, ****p = 0.0003. No significant effect of treatment [*F*(2,29) = 0.06, *p* = 0.94] or interaction [*F*(2,29) = 2.442, *p* = 0.10]. Sidak’s multiple comparisons test sed young vs. aged *t*(8) = 0.43, *p* = 0.96; ex young vs. aged *t*(10) = 3.45, **p = 0.0052; etp young vs. aged *t*(11) = 3.79, **p = 0.0021.

Our lab previously found that H3K9me3 is increased with age in the hippocampi of aged mice, leading us to ask if promoter bound H3K9me3 was also elevated with age in our study (Snigdha et al., 2016). We compared percent input levels from ChIP experiments measuring H3K9me3 binding in young and aged mice. Surprisingly, we found that H3K9me3 promoter binding was greater in young mice than in aged mice at BDNF promoters I, IV, and VI, as well as at the GABRA2 promoter (Figures 6A,B,C,E). There was no effect of age on H3K9me3 binding at the GABBR1 promoter (Figure 6D). This finding suggests that H3K9me3 promoter repression is impaired with increasing age.

## Discussion

Our findings demonstrate that exercise and SUV39H1 inhibition affect the expression of BDNF exons and GABRA2 in an age dependent manner. Exercise and ETP69 significantly decreased H3K9me3 at BDNF promoter VI in aged mice, corresponding to an increase in BDNF VI expression with ETP69 treatment. Apart from this important exception, exercise and ETP69 did not significantly alter H3K9me3 levels at the promoters of neuronal plasticity genes, although they did regulate mRNA levels. These findings suggest that H3K9me3 promoter repression is not a primary driver of neuronal plasticity gene expression. Age had a strong effect on H3K9me3 promoter binding, as H3K9me3 repression of BDNF and GABA receptors decreased significantly in old mice. Exercise and ETP69 altered neuronal gene expression in an age-dependent manner and reduced H3K9me3 recruitment at the BDNF VI promoter region.

Of the promoters we examined, exercise and ETP69 only reduced H3K9me3 at the BDNF VI promoter in aged mice, suggesting that promoter bound H3K9me3 is a largely stable epigenetic modification. This finding differs from previous research showing that ETP69 reduces H3K9me3 at BDNF I following an OLM task (Snigdha et al., 2016). OLM increases the expression of the immediate early gene Nr4a2, which activates BDNF promoters and modulates BDNF expression (Volpicelli et al., 2007; Kwapis et al., 2019). Inhibiting SUV39H1 thus had a disparate effect on BDNF regulation in home cage mice as opposed to cognitively engaged mice. ETP69 increased BDNF VI expression in aged mice, potentially as a means of improving plasticity in a system where synaptic genes are downregulated (Lu et al., 2004). BDNF VI is localized to distal dendrites while BDNF IV is restricted to the soma, and BDNF I is present in both soma and dendrites (Pattabiraman et al., 2005; Chiaruttini et al., 2009; Boulle et al., 2012). BDNF VI is also involved in governing the complexity of dendritic spines (Maynard et al., 2017). This regulatory exon may be selectively upregulated by SUV39H1 inhibition to improve plasticity in the aged brain.

In addition to increasing BDNF VI levels in aged mice, exercise also elevated BDNF I expression in young mice. This finding is supported by previous studies in our lab showing that 3 weeks of wheel running increase BDNF I levels in the hippocampi of young rats and mice (Tong et al., 2001; Intlekofer et al., 2013). There was no change in BDNF IV in young or aged mice, a finding which is supported by an exercise study in young mice (Intlekofer et al., 2013). As previously mentioned, BDNF IV is localized at the soma as opposed to dendrites like BDNF I and VI and does not play a significant role in synaptic plasticity (Pattabiraman et al., 2005; Chiaruttini et al., 2009; Boulle et al., 2012). BDNF IV may also be more involved in fear and emotion based learning than BDNF I and VI. A fear conditioning study showed that consolidation of fear learning upregulated BDNF IV expression, while context exposure alone was enough to upregulate BDNF I and VI (Lubin et al., 2008; Boulle et al., 2012). Based on these studies, it is possible that BDNF IV expression is not regulated by the same mechanism as BDNF I and VI.

Intriguingly, exercise and SUV39H1 inhibition significantly affected GABRA2 expression, yet the direction of change was dependent on mouse age. While exercise increased GABRA2 expression in aged mice, ETP69 and exercise both reduced GABRA2 levels in young mice. GABA signaling is reduced in the hippocampus with age, contributing to overexcitation and memory impairment (Stanley and Shetty, 2004; Spiegel et al., 2013; McQuail et al., 2015). A study of 21-month-old mice found a specific decrease in GABRA2 around neuronal cell bodies and proximal dendrites in the CA1 and CA3 (Palpagama et al., 2019). GABA A receptor activity is also correlated with cognitive performance in aged mice (Koh et al., 2013, 2020). Exercise may induce an increase in GABRA2 in the aged hippocampus to compensate for an age-associated decline in inhibitory signaling, improving cognition in the process. In contrast, exercise may reduce GABA receptors in young mice to increase neuronal excitation. This is supported by a study showing that 4 weeks of wheel running decreased GABRA2 in the forebrain of young rats (Hill et al., 2010). GABRA2 expression was not mediated by promoter bound H3K9me3, suggesting that repression of upstream transcriptional elements may play a greater role in regulating gene expression. H3K9me3 is prevalent in regions flanking enhancer regions, suggesting that exercise may reduce enhancer H3K9me3 in aged mice and increase repression in young mice (Zhu et al., 2012). Conversely, ETP69 may decrease GABRA2 in young mice by reducing H3K9me3 at transcriptional suppressors. Exercise and SUV39H1 inhibition can dynamically impact neuronal plasticity, but the pathway by which they regulate neuronal gene expression remains to be established.

Exercise induced changes in BDNF and GABRA2 expression in our models, yet GABRA2’s role in BDNF regulation remains unclear. In aged and exercised mice, the increase in GABRA2 correlated with an increase in BDNF VI expression, suggesting that GABRA2 may upregulate BDNF in aged models of exercise. In contrast, exercise induced a significant decrease in GABRA2 and an increase in BDNF I expression in young mice. GABRA2 may only be involved in BDNF upregulation in systems where BDNF and GABA signaling is impaired, such as models of aging. Future studies should examine the signaling pathway between GABRA2 and BDNF in models of aging and exercise to determine if GABRA2 differentially upregulates BDNF expression.

Although promoter H3K9me3 was largely unaffected by our manipulations, its stability and binding affinity appear to decline with age. With the exception of GABBR1, H3K9me3 binding was greater in young mice than aged mice at the promoters of neuronal plasticity genes. Our findings demonstrate that H3K9me3 at promoter regions decreases with age even as overall H3K9me3 increases, which may indicate a change in H3K9me3’s localization and function (Snigdha et al., 2016). Although we examined promoter bound H3K9me3 in this study, H3K9me3 is more abundant at repeat rich sequences packaged into constitutive heterochromatin (Maze et al., 2011; Becker et al., 2017). These regions may be under tighter control with age as increased age is associated with impaired heterochromatin stability (Kane and Sinclair, 2019). Chronic stress has also been linked to chromatin remodeling, and the stress associated with advanced age may similarly change H3K9me3 localization (Hunter et al., 2009). Apart from gene repression and heterochromatin maintenance, H3K9me3 is also involved in double strand break repair (Ayrapetov et al., 2014). Future studies should explore if the genomic placement and function of H3K9me3 changes with age.

This study opens up avenues for future investigation into the regulation of neuronal plasticity genes. The role of GABRA2 in BDNF regulation has yet to be explored, and studies involving GABRA2 inhibition or overexpression could elucidate its effect on BDNF expression. Studies on H3K9me3 localization throughout the lifespan will further suggest how its function changes with age. Our SUV39H1 inhibitor, ETP69, globally reduces H3K9me3 but cannot modify this marker at specific genes. ChIP-sequencing could be used to identify how H3K9me3 binding changes with age and exercise, and RNA-sequencing can determine if these changes are correlated with gene expression. A comprehensive examination of H3K9me3 will clarify its effect on neuronal gene expression and specific sites of pharmacological manipulation.

## Conclusion

We have demonstrated that H3K9me3 regulates the expression of BDNF VI in aged mice. Reducing H3K9me3 with ETP69 increases BDNF VI expression, thus increasing synaptic plasticity at distal dendrites. Increased age reduces H3K9me3 promoter binding and changes the regulation of BDNF and GABRA2 by exercise and ETP69. Overall, exercise and SUV39H1 inhibition can effectively modify the expression of genes involved in cognition.

## Data Availability Statement

The original contributions presented in the study are included in the article/Supplementary Material, further inquiries can be directed to the corresponding author.

## Ethics Statement

The animal study was reviewed and approved by the Institutional Animal Care and Use Committee of the University of California, Irvine.

## Author Contributions

AI-T and NB conceived of the presented ideas. CB and AI-T performed the experiments. DM and MW assisted with experimental design and provided PCR equipment. AI-T analyzed the data and wrote the manuscript. CC reviewed the manuscript and supervised the project. All authors contributed to the article and approved the submitted version.

## Conflict of Interest

The authors declare that the research was conducted in the absence of any commercial or financial relationships that could be construed as a potential conflict of interest.

## Publisher’s Note

All claims expressed in this article are solely those of the authors and do not necessarily represent those of their affiliated organizations, or those of the publisher, the editors and the reviewers. Any product that may be evaluated in this article, or claim that may be made by its manufacturer, is not guaranteed or endorsed by the publisher.

## References

[B1] Adlard P. A. Engesser-Cesar C. Cotman C. W. (2011). Mild stress facilitates learning and exercise improves retention in aged mice. *Exp. Gerontol.* 46 53–59. 10.1016/j.exger.2010.10.001 20951791

[B2] Adlard P. A. Perreau V. M. Cotman C. W. (2005). The exercise-induced expression of BDNF within the hippocampus varies across life-span. *Neurobiol. Aging* 26 511–520. 10.1016/j.neurobiolaging.2004.05.006 15653179

[B3] Adlard P. A. Perreau V. M. Engesser-Cesar C. Cotman C. W. (2004). The timecourse of induction of brain-derived neurotrophic factor mRNA and protein in the rat hippocampus following voluntary exercise. *Neurosci. Lett.* 363 43–48. 10.1016/j.neulet.2004.03.058 15157993

[B4] Aid T. Kazantseva A. Piirsoo M. Palm K. Timmusk T. (2007). Mouse and rat BDNF gene structure and expression revisited. *J. Neurosci. Res.* 85 525–535. 10.1002/jnr.21139 17149751 PMC1878509

[B5] Ayrapetov M. K. Gursoy-Yuzugullu O. Xu C. Xu Y. Price B. D. (2014). DNA double-strand breaks promote methylation of histone H3 on lysine 9 and transient formation of repressive chromatin. *Proc. Natl. Acad. Sci. U S A.* 111 9169–9174. 10.1073/pnas.1403565111 24927542 PMC4078803

[B6] Baumann M. Dieskau A. P. Loertscher B. M. Walton M. C. Nam S. Xie J. (2015). Tricyclic Analogues of Epidithiodioxopiperazine Alkaloids with Promising. *Chem. Sci.* 6 4451–4457. 10.1039/C5SC01536G 26301062 PMC4540405

[B7] Becker J. S. McCarthy R. L. Sidoli S. Donahue G. Kaeding K. E. He Z. (2017). Genomic and Proteomic Resolution of Heterochromatin and Its Restriction of Alternate Fate Genes. *Mol. Cell* 68 1023.e–1037.e. 10.1016/j.molcel.2017.11.030 29272703 PMC5858919

[B8] Berchtold N. C. Kesslak J. P. Cotman C. W. (2002). Hippocampal brain-derived neurotrophic factor gene regulation by exercise and the medial septum. *J. Neurosci. Res.* 68 511–521. 10.1002/jnr.10256 12111841

[B9] Berchtold N. C. Prieto G. A. Phelan M. Gillen D. L. Baldi P. Bennett D. A. (2019). Hippocampal gene expression patterns linked to late-life physical activity oppose age and AD-related transcriptional decline. *Neurobiol. Aging* 78 142–154. 10.1016/j.neurobiolaging.2019.02.012 30927700 PMC6901108

[B10] Boulle F. van den Hove D. L. Jakob S. B. Rutten B. P. Hamon M. van Os J. (2012). Epigenetic regulation of the BDNF gene: implications for psychiatric disorders. *Mol. Psychiatry* 17 584–596. 10.1038/mp.2011.107 21894152

[B11] Brady M. L. Pilli J. Lorenz-Guertin J. M. Das S. Moon C. E. Graff N. (2018). Depolarizing, inhibitory GABA type A receptor activity regulates GABAergic synapse plasticity via ERK and BDNF signaling. *Neuropharmacology* 128 324–339. 10.1016/j.neuropharm.2017.10.022 29074304 PMC5739058

[B12] Chiaruttini C. Vicario A. Li Z. Baj G. Braiuca P. Wu Y. (2009). Dendritic trafficking of BDNF mRNA is mediated by translin and blocked by the G196A (Val66Met) mutation. *Proc. Natl. Acad. Sci. U S A.* 106 16481–16486. 10.1073/pnas.0902833106 19805324 PMC2752540

[B13] Cotman C. W. Berchtold N. C. (2002). Exercise: a behavioral intervention to enhance brain health and plasticity. *Trends Neurosci.* 25 295–301. 10.1016/s0166-2236(02)02143-4 12086747

[B14] Fernandes J. Arida R. M. Gomez-Pinilla F. (2017). Physical exercise as an epigenetic modulator of brain plasticity and cognition. *Neurosci. Biobehav. Rev.* 80 443–456. 10.1016/j.neubiorev.2017.06.012 28666827 PMC5705447

[B15] Fiorentino H. Kuczewski N. Diabira D. Ferrand N. Pangalos M. N. Porcher C. (2009). GABA(B) receptor activation triggers BDNF release and promotes the maturation of GABAergic synapses. *J. Neurosci.* 29 11650–11661. 10.1523/JNEUROSCI.3587-09.2009 19759312 PMC6665767

[B16] Hill L. E. Droste S. K. Nutt D. J. Linthorst A. C. Reul J. M. (2010). Voluntary exercise alters GABA(A) receptor subunit and glutamic acid decarboxylase-67 gene expression in the rat forebrain. *J. Psychopharmacol.* 24 745–756. 10.1177/0269881108096983 18801833

[B17] Hunter R. G. McCarthy K. J. Milne T. A. Pfaff D. W. McEwen B. S. (2009). Regulation of hippocampal H3 histone methylation by acute and chronic stress. *Proc. Natl. Acad. Sci. U S A.* 106 20912–20917. 10.1073/pnas.0911143106 19934035 PMC2791599

[B18] Intlekofer K. A. Berchtold N. C. Malvaez M. Carlos A. J. McQuown S. C. Cunningham M. J. (2013). Exercise and sodium butyrate transform a subthreshold learning event into long-term memory via a brain-derived neurotrophic factor-dependent mechanism. *Neuropsychopharmacology* 38 2027–2034. 10.1038/npp.2013.104 23615664 PMC3746687

[B19] Kane A. E. Sinclair D. A. (2019). Epigenetic changes during aging and their reprogramming potential. *Crit. Rev. Biochem. Mol. Biol.* 54 61–83. 10.1080/10409238.2019.1570075 30822165 PMC6424622

[B20] Kirk-Sanchez N. J. McGough E. L. (2014). Physical exercise and cognitive performance in the elderly: current perspectives. *Clin. Interv. Aging* 9 51–62. 10.2147/CIA.S39506 24379659 PMC3872007

[B21] Koh M. T. Branch A. Haberman R. Gallagher M. (2020). Significance of inhibitory recruitment in aging with preserved cognition: limiting gamma-aminobutyric acid type A α5 function produces memory impairment. *Neurobiol. Aging* 91 1–4. 10.1016/j.neurobiolaging.2020.02.019 32240868 PMC7641038

[B22] Koh M. T. Rosenzweig-Lipson S. Gallagher M. (2013). Selective GABA(A) α5 positive allosteric modulators improve cognitive function in aged rats with memory impairment. *Neuropharmacology* 64 145–152. 10.1016/j.neuropharm.2012.06.023 22732440 PMC3445657

[B23] Kolarow R. Brigadski T. Lessmann V. (2007). Postsynaptic secretion of BDNF and NT-3 from hippocampal neurons depends on calcium calmodulin kinase II signaling and proceeds via delayed fusion pore opening. *J. Neurosci.* 27 10350–10364. 10.1523/JNEUROSCI.0692-07.2007 17898207 PMC6673152

[B24] Kwapis J. L. Alaghband Y. López A. J. Long J. M. Li X. Shu G. (2019). HDAC3-Mediated Repression of the *Nr4a* Family Contributes to Age-Related Impairments in Long-Term Memory. *J. Neurosci.* 39 4999–5009. 10.1523/JNEUROSCI.2799-18.2019 31000586 PMC6670247

[B25] Lardenoije R. Iatrou A. Kenis G. Kompotis K. Steinbusch H. W. Mastroeni D. (2015). The epigenetics of aging and neurodegeneration. *Prog. Neurobiol.* 131 21–64. 10.1016/j.pneurobio.2015.05.002 26072273 PMC6477921

[B26] Lee M. Y. Lee J. Hyeon S. J. Cho H. Hwang Y. J. Shin J.-Y. (2020). Epigenome signatures landscaped by histone H3K9me3 are associated with the synaptic dysfunction in Alzheimer’s disease. *Aging Cell* 19:e13153. 10.1111/acel.13153 32419307 PMC7294781

[B27] Lu T. Pan Y. Kao S. Y. Li C. Kohane I. Chan J. (2004). Gene regulation and DNA damage in the ageing human brain. *Nature* 429 883–891. 10.1038/nature02661 15190254

[B28] Lubin F. D. Roth T. L. Sweatt J. D. (2008). Epigenetic regulation of BDNF gene transcription in the consolidation of fear memory. *J. Neurosci.* 28 10576–10586. 10.1523/JNEUROSCI.1786-08.2008 18923034 PMC3312036

[B29] Malvaez M. Mhillaj E. Matheos D. P. Palmery M. Wood M. A. (2011). CBP in the nucleus accumbens regulates cocaine-induced histone acetylation and is critical for cocaine-associated behaviors. *J. Neurosci.* 31 16941–16948. 10.1523/JNEUROSCI.2747-11.2011 22114264 PMC3235434

[B30] Maynard K. R. Hobbs J. W. Sukumar M. Kardian A. S. Jimenez D. V. Schloesser R. J. (2017). Bdnf mRNA splice variants differentially impact CA1 and CA3 dendrite complexity and spine morphology in the hippocampus. *Brain Struct. Funct.* 222 3295–3307. 10.1007/s00429-017-1405-3 28324222 PMC5608635

[B31] Maze I. Feng J. Wilkinson M. B. Sun H. Shen L. Nestler E. J. (2011). Cocaine dynamically regulates heterochromatin and repetitive element unsilencing in nucleus accumbens. *Proc. Natl. Acad. Sci. U S A.* 108 3035–3040. 10.1073/pnas.1015483108 21300862 PMC3041122

[B32] McQuail J. A. Frazier C. J. Bizon J. L. (2015). Molecular aspects of age-related cognitive decline: the role of GABA signaling. *Trends Mol. Med.* 21 450–460. 10.1016/j.molmed.2015.05.002 26070271 PMC4500156

[B33] Neeper S. A. Gómez-Pinilla F. Choi J. Cotman C. (1995). Exercise and brain neurotrophins. *Nature* 373:109. 10.1038/373109a0 7816089

[B34] Palpagama T. H. Sagniez M. Kim S. Waldvogel H. J. Faull R. L. Kwakowsky A. (2019). GABA A Receptors Are Well Preserved in the Hippocampus of Aged Mice. *eNeuro* 6:2019. 10.1523/ENEURO.0496-18.2019 31340951 PMC6709233

[B35] Pattabiraman P. P. Tropea D. Chiaruttini C. Tongiorgi E. Cattaneo A. Domenici L. (2005). Neuronal activity regulates the developmental expression and subcellular localization of cortical BDNF mRNA isoforms in vivo. *Mol. Cell Neurosci.* 28 556–570. 10.1016/j.mcn.2004.11.010 15737745

[B36] Pfaffl M. W. (2001). A new mathematical model for relative quantification in real-time RT-PCR. *Nucleic Acids Res.* 29:e45. 10.1093/nar/29.9.e45 11328886 PMC55695

[B37] Porcher C. Medina I. Gaiarsa J. L. (2018). Mechanism of BDNF Modulation in GABAergic Synaptic Transmission in Healthy and Disease Brains. *Front. Cell Neurosci.* 12:273. 10.3389/fncel.2018.00273 30210299 PMC6121065

[B38] Rogge G. A. Singh H. Dang R. Wood M. A. (2013). HDAC3 is a negative regulator of cocaine-context-associated memory formation. *J. Neurosci.* 33 6623–6632. 10.1523/JNEUROSCI.4472-12.2013 23575859 PMC3670682

[B39] Sedivy J. M. Banumathy G. Adams P. D. (2008). Aging by epigenetics–a consequence of chromatin damage? *Exp. Cell Res.* 314 1909–1917. 10.1016/j.yexcr.2008.02.023 18423606 PMC2464300

[B40] Snigdha S. de Rivera C. Milgram N. W. Cotman C. W. (2014). Exercise enhances memory consolidation in the aging brain. *Front. Aging Neurosci.* 6:3. 10.3389/fnagi.2014.00003 24550824 PMC3910002

[B41] Snigdha S. Prieto G. A. Petrosyan A. Loertscher B. M. Dieskau A. P. Overman L. E. (2016). H3K9me3 Inhibition Improves Memory, Promotes Spine Formation, and Increases BDNF Levels in the Aged Hippocampus. *J. Neurosci.* 36 3611–3622. 10.1523/JNEUROSCI.2693-15.2016 27013689 PMC4804016

[B42] Spiegel A. M. Koh M. T. Vogt N. M. Rapp P. R. Gallagher M. (2013). Hilar interneuron vulnerability distinguishes aged rats with memory impairment. *J. Comp. Neurol.* 521 3508–3523. 10.1002/cne.23367 23749483 PMC4801143

[B43] Stanley D. P. Shetty A. K. (2004). Aging in the rat hippocampus is associated with widespread reductions in the number of glutamate decarboxylase-67 positive interneurons but not interneuron degeneration. *J. Neurochem.* 89 204–216. 10.1111/j.1471-4159.2004.02318.x 15030405

[B44] Tong L. Shen H. Perreau V. M. Balazs R. Cotman C. W. (2001). Effects of exercise on gene-expression profile in the rat hippocampus. *Neurobiol. Dis.* 8 1046–1056. 10.1006/nbdi.2001.0427 11741400

[B45] van Praag H. Shubert T. Zhao C. Gage F. H. (2005). Exercise enhances learning and hippocampal neurogenesis in aged mice. *J. Neurosci.* 25 8680–8685. 10.1523/JNEUROSCI.1731-05.2005 16177036 PMC1360197

[B46] Villeneuve L. M. Kato M. Reddy M. A. Wang M. Lanting L. Natarajan R. (2010). Enhanced levels of microRNA-125b in vascular smooth muscle cells of diabetic db/db mice lead to increased inflammatory gene expression by targeting the histone methyltransferase Suv39h1. *Diabetes* 59 2904–2915. 10.2337/db10-0208 20699419 PMC2963550

[B47] Volpicelli F. Caiazzo M. Greco D. Consales C. Leone L. Perrone-Capano C. (2007). Bdnf gene is a downstream target of Nurr1 transcription factor in rat midbrain neurons in vitro. *J. Neurochem.* 102 441–453. 10.1111/j.1471-4159.2007.04494.x 17506860

[B48] Zhu Y. van Essen D. Saccani S. (2012). Cell-type-specific control of enhancer activity by H3K9 trimethylation. *Mol. Cell* 46 408–423. 10.1016/j.molcel.2012.05.011 22633489

