# Supplementary material for: Exercise Reduces H3K9me3 and Regulates Brain Derived Neurotrophic Factor and GABRA2 in an Age Dependent Manner
Source: Front Aging Neurosci. 2021 Dec 14;13:798297. doi: 10.3389/fnagi.2021.798297 (PMC8712855; doi:10.3389/fnagi.2021.798297)
Supplement: Supplementary file 1 [file Table_1.docx]

Supplementary Material

**Supplementary Table 1 qPCR Primer Sequences for ChIP**

| **Promoter and Refseq Accession Number** | **Primer Sequence 5’-3’** | **Corresponding to Position** | **Amplicon (bp)** |
| --- | --- | --- | --- |
| BDNF 1  NT_039207 | TGATCATCACTCACGACCACG | 50555972-50555992 | 134 |
|  | CAGCCTCTCTGAGCCAGTTAC | 50556105-50556085 |  |
| BDNF IV  NT_039207 | TGCGCGGAATTCTGATTCTGG | 50573782-50573802 | 107 |
|  | GTCCACGAGAGGGCTCCACG | 50573888-50573871 |  |
| BDNF VI  NT_039207 | ACTCACACTCGCTTCCTCCT | 50574691-50574710 | 171 |
|  | GCACTGGCTTCTCTCCATTT | 50574861-50574842 |  |
| GABBR1  NC_000083.7 | CAGGAGAGCGAAAGGGGAAG | 37383295-37383314 | 299 |
|  | AACAGCGCCAAGAGAATGGA | 37383593-37383574 |  |
| GABRA2  NC_000071.7 | TTCTGGGGAGGGACATTGGA | 71252739-71252758 | 141 |
|  | TGCTCATTCCCCTCTGCTTC | 71252879-71252860 |  |
