# Supplementary material for: Exercise Reduces H3K9me3 and Regulates Brain Derived Neurotrophic Factor and GABRA2 in an Age Dependent Manner
Source: Front Aging Neurosci. 2021 Dec 14;13:798297. doi: 10.3389/fnagi.2021.798297 (PMC8712855; doi:10.3389/fnagi.2021.798297)
Supplement: Supplementary file 2 [file Table_2.docx]

Supplementary Material

**Supplementary Table 2 qPCR Primer and Probe Sequences for mRNA**

| **Gene Name and Refseq Accession Number** | **Primer and Probe Sequence 5’-3’** | **Corresponding to Position** | **Amplicon (bp)** |
| --- | --- | --- | --- |
| BDNF I  NM_007540.4 | GACACATTACCTTCCTGCATCT | 564-586 | 108 |
|  | GGATGGTCATCACTCTTCTCAC | 650-629 |  |
|  | Probe: ACAGCAAAGCCACAATGTTCCACC | 624-648 |  |
| BDNF IV  NM_001048141.1 | TCCCCTTCTCTTCAGTTAAAAGG | 177-197 | 73 |
|  | TTGCTGCAGAACAGGACTACA | 249-227 |  |
|  | Probe: TATCGGCCACCAAAGACTCGCC | 198-219 |  |
| BDNF VI  NM_001048142.1 | CTGAGCGTGTGTGACAGTATTA | 786-808 | 112 |
|  | CTTTGGATACCGGGACTTTCTC | 876-898 |  |
|  | Probe:TTTATCTGCCGCTGTGACCCACTC | 810-834 |  |
| GABBR1  NM_019439.3 | GCTCTTGGGCTTAGGCTTTA | 2325-2345 | 109 |
|  | GGTCTTCCTCCATTCCTTCTTC | 2412-2434 |  |
|  | Probe:TTGTGAAGACTGTGTGGACCCACC | 2378-2402 |  |
| GABRA2  NM_008066.4 | AGAGAGAGCTCAGAGGATAACA | 4107-4129 | 115 |
|  | CAGAGCTCCTACAGCACATATC | 4200-4222 |  |
|  | Probe:AGAGGGCGTGATTTCAAGTTCCCA | 4146-4170 |  |
| GAPDH  NM_008084.3 | AATGGTGAAGGTCGGTGTG | 240-258 | 150 |
|  | GTGGAGTCATACTGGAACATGTAG | 389-366 |  |
|  | Probe:TGCAAATGGCAGCCCTGGTG | 307-288 |  |
